# The First Molecular Characterization of Serbian SARS-CoV-2 Isolates From a Unique Early Second Wave in Europe

**DOI:** 10.3389/fmicb.2021.691154

**Published:** 2021-06-18

**Authors:** Danijela Miljanovic, Ognjen Milicevic, Ana Loncar, Dzihan Abazovic, Dragana Despot, Ana Banko

**Affiliations:** ^1^Virology Laboratory, Faculty of Medicine, Institute of Microbiology and Immunology, University of Belgrade, Belgrade, Serbia; ^2^Faculty of Medicine, Institute for Medical Statistics and Informatics, University of Belgrade, Belgrade, Serbia; ^3^Laboratory of Molecular Microbiology, Institute for Biocides and Medical Ecology, Belgrade, Serbia; ^4^Biocell Hospital, Belgrade, Serbia; ^5^Emergency Medical Centre of Montenegro, Podgorica, Montenegro

**Keywords:** SARS-CoV-2, COVID-19, Serbia, NGS, genetic diversity

## Abstract

March 6, 2020 is considered as the official date of the beginning of the COVID-19 epidemic in Serbia. In late spring and early summer 2020, Europe recorded a decline in the rate of SARS-CoV-2 infection and subsiding of the first wave. This trend lasted until the fall, when the second wave of the epidemic began to appear. Unlike the rest of Europe, Serbia was hit by the second wave of the epidemic a few months earlier. Already in June 2020, newly confirmed cases had risen exponentially. As the COVID-19 pandemic is the first pandemic in which there has been instant sharing of genomic information on isolates around the world, the aim of this study was to analyze whole SARS-CoV-2 viral genomes from Serbia, to identify circulating variants/clade/lineages, and to explore site-specific mutational patterns in the unique early second wave of the European epidemic. This analysis of Serbian isolates represents the first publication from Balkan countries, which demonstrates the importance of specificities of local transmission especially when preventive measures differ among countries. One hundred forty-eight different genome variants among 41 Serbian isolates were detected in this study. One unique and seven extremely rare mutations were identified, with locally specific continuous dominance of the 20D clade. At the same time, amino acid substitutions of newly identified variants of concern were found in our isolates from October 2020. Future research should be focused on functional characterization of novel mutations in order to understand the exact role of these variations.

## Introduction

*“Everything that happens once can never happen again. But everything that happens twice will surely happen a third time.”* (Paulo Coelho, *The Alchemist*).

Coronaviruses have emerged three times in the twenty-first century, causing a worldwide epidemic and pandemic. The first case of coronavirus disease 2019 (COVID-19) was reported in December 2019 in Wuhan City, Hubei Province, China (Zhou et al., 2020). In a short time, it has spread around the world and has been declared one of the largest pandemics to date. By the end of April 2021, more than 150 million individuals had been affected, leading to more than 3 million deaths. Since 6 March, 2020, sustained local transmission of COVID-19 has been documented in Serbia, where to date (May 2021, almost 686,000 cases positive for Severe Acute Respiratory Disease Coronavirus 2 (SARS-CoV-2) have been diagnosed, with more than 6,300 deaths. Unlike the rest of Europe, which was hit by the second wave of the pandemic in the fall of 2020, the local outbreak in Serbia happened significantly earlier, at the beginning of the summer.

COVID-19 is caused by a novel virus, SARS-CoV-2 which belongs to the family *Coronaviridae*, subfamily *Coronavirinae*, and genera beta-coronavirus. All the CoVs are enveloped, positive-sense, single-stranded RNA viruses with a polycistronic genome approximately 30 kb in length, which represents the largest genome among all RNA viruses (Wu F. et al., 2020; Zhou et al., 2020). The SARS-CoV-2 genome consists of a 5′ untranslated region (5′UTR), genes for structural and non-structural proteins, several open reading frames encoding accessory proteins, and a 3′UTR (Kim J. S. et al., 2020). The structural proteins include spike (S), envelope (E), membrane (M), and nucleocapsid (N) protein. Of all structural elements on the surface, the protruding trimeric S protein plays a vital role during viral entry, using angiotensin-converting enzyme 2 (ACE2) as a receptor on human cells (Li et al., 2003). Similar to other coronaviruses, the SARS-CoV-2 genome contains several open reading frames (ORFs). One of them, ORF1ab, is proteolytically cleaved into 16 non-structural proteins (nsp1–nsp16) that are essential for viral replication and immune evasion (Snijder et al., 2003; Cagliani et al., 2020). Other open reading frames, located in between genes for structural proteins, define accessory proteins: ORF3a, ORF6, ORF7a, ORF7b, ORF8, and ORF10 (Almubaid and Al-Mubaid, 2021).

Because SARS-CoV-2 has an intrinsic proofreading ability to correct mistakes made during replication (Sanjuán and Domingo-Calap, 2016), it does not show a high mutation rate like other RNA viruses. However, the excessive number of infected hosts combined with duration of pandemic leads to fast spread of virus variants with enhanced fitness and replication potential. Up to May 2020, more than million SARS-CoV-2 whole genome sequences were available on the Global Initiative on Sharing All Influenza Data (GISAID)^1^. The number of sequences has rapidly increased in the last few months thanks to extraordinary efforts of the global scientific community. In the last months of 2020, many parts of the world were just beginning mass sequencing and providing missing data. In addition to the limited number of available SARS-CoV-2 sequences from Balkan countries, a study that offers detailed analysis of local sequences has not yet been published. Monitoring of all local SARS-CoV-2 genomic diversity represents a necessary element for understanding global viral evolution. In order to trace infection pathways, predict future trajectories for virulence and transmissibility, and to create preventive strategies for COVID-19, it is necessary to identify key mutations in SARS-CoV-2 genome as they emerge and spread. Moreover, it has been suggested that the current SARS-CoV-2 reference genome should be re-evaluated, even replaced with a new one that represents the viral population more accurately (Urhan and Abeel, 2021).

The aim of this study was to analyze whole SARS-CoV-2 viral genomes from Serbia, to identify circulating variants/clade/lineages, and to explore site-specific mutational patterns in the second wave of COVID-19 epidemic in Serbia that took place in summer 2020.

## Materials and Methods

### COVID-19 Patients and Samples

Beginning April 2020, the Laboratory of Molecular Microbiology, Institute for Biocides and Medical Ecology, Belgrade, was designated as one of the relevant laboratories for COVID-19 research. During this period, nasopharyngeal and oropharyngeal swabs from patients from different regions of Serbia were taken according to World Health Organization, WHO guidelines (World Health Organization [WHO], 2020), immersed in 3 ml of viral transport medium (Liuyang SANLI Medical Technology), and submitted for molecular diagnosis for SARS-CoV-2. In this study, 41 randomly selected positive samples collected from June to October 2020 with a wide territorial distribution were used for whole genome sequencing. Thirty samples were collected from hospitalized patient and 11 originated from non-hospitalized individuals which were tested in mentioned laboratory. The inclusion criteria also included Ct values bellow 25.

### Extraction and Real-Time PCR

RNA extraction from 500 μl viral transport medium was performed using RNeasy Mini Kit (Qiagen). Real-time PCR for confirmation of SARS-CoV-2 positivity was performed with 5 μl of eluted RNA following the manufacturer’s protocol, using GeneFinder COVID/19 Plus Real*Amp* Kit. The limiting factor for sample selection was a real-time PCR Ct value below 25.

### SARS-CoV-2 Next Generation Sequencing (NGS)

Sequencing was commercially done at the Access To Genome Clinical Laboratory Genetics (Access to Genome, Clinical Laboratory Genetics, 55134 Thessaloniki, Greece) with “Ion Torrent” (Thermo Scientific) technology. The instructions for Ion Ampliseq SARS-CoV-2 Research Panel with Ion Ampliseq Library kit 2.0 were followed for library construction. Briefly, two pools of amplicons of length 125–275 bp (mean 199 bp) were used covering >99% for all viral serotypes. An Ion Chef Instrument was used for template preparation and the I on 550 chip was run on the Ion GeneStudio^TM^ S5 Prime System. Median depth of coverage for all the sequences is 2,123 (range 19–5,950).

### Bioinformatics Analysis

The alignment and analysis of the samples was performed on Torrent Server–Torrent Suite (version 5.16). Variant identification and annotation were performed running the COVID19AnnotateSnpEff plugin. The FASTA sequences containing the SARS-CoV-2 genome were generated running the IRMA report plugin.

Additional filtering was performed based on allele zygosity and quality. Heterozygous sites with quality below 300 were rejected while those qualities between 300 and 500 were manually curated in a genome browser.

### Phylogenetic Analysis

The lineages of Serbian SARS-CoV-2 isolates were determined using the Pangolin COVID 19 Lineage Assigner Interface^2^ (Rambaut et al., 2020). Clades were assigned by using GISAID platform and also by the Nextclade sequence analysis webapp^3^ (Hadfield et al., 2018). The mutation analyses of SARS-CoV-2 isolates from this study were determined by comparison to the reference hCoV-19/Wuhan/wiv04/2019 strain by using the GISAID database feature CoVsurver: Mutation Analysis^4^.

A total of 41 whole genome sequences of SARS-CoV-2 were obtained in this study and all sequences from neighboring Balkan countries (Albania, Bosnia and Herzegovina, North Macedonia, Montenegro, Slovenia, and Croatia) reported in period June–October 2020 were retrieved from the GISAID database (*n* = 160) together with the reference sequence Wuhan-Hu-1 NC 045512.2. They were aligned with the CLUSTAL W program implemented in MEGA X: Molecular Evolutionary Genetics Analysis across computing platforms (Kumar et al., 2018). The most appropriate model of evolution for this region was inferred using jModelTest 0.1.1 (Posada, 2008). The maximum-likelihood (ML) tree was created by the W-IQ-TREE web server^5^ with 10,000 ultrafast bootstraps (Trifinopoulos et al., 2016; Hoang et al., 2018) using the previously defined best-fit GTR^+^ G model of evolution. The tree obtained as output from the IQTREE software was opened in FigTree 1.3.1^6^ for initial viewing. The tree was exported from FigTree 1.3.1 into MEGA X for labeling and additional notations.

## Results

### Distribution of SARS-CoV-2 Lineages and Clades in Isolates From the Second Wave of the Epidemic in Serbia (June–October 2020)

**FIGURE 1:**
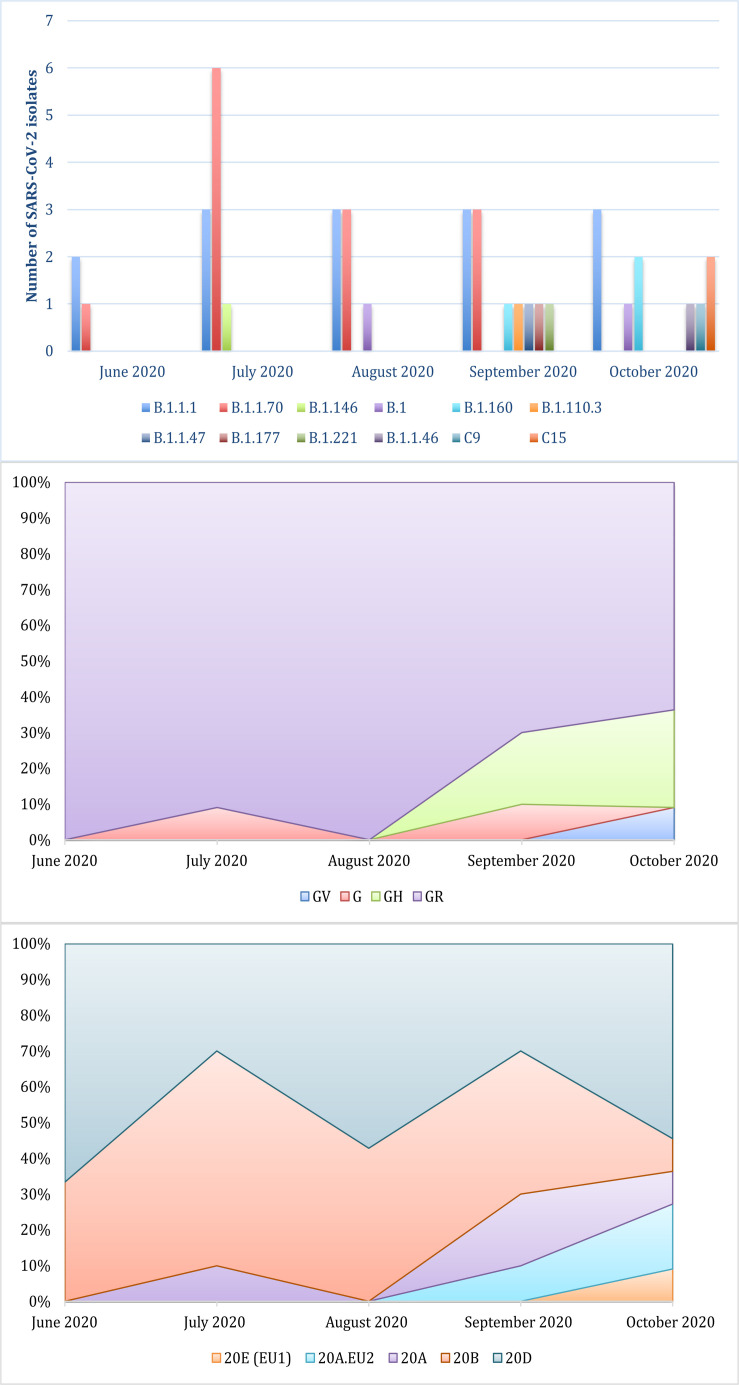
Distribution of lineages and clades of SARS-CoV-2 isolates from the Serbian second wave assigned by PANGOLIN, GISAID, and NEXTRAIN from the top to the bottom.

This study focused on the genomic characterization of 41 SARS-CoV-2 whole genome sequences from the second wave of the epidemic in Serbia. Among currently available naming systems for division of SARS-CoV-2 isolates into distinct variants, clades, and lineages, three dynamic platforms have been established as the most accepted: PANGO, GISAID, and NEXTRAIN. This prevents the uniformity and clarity that exists for other pathogens. Because of that, a universal naming system is urgently needed. According to PANGO nomenclature, all Serbian sequences belonged to two parent lineages, B and C. Further lineage analysis resulted in the assignment of each isolate to 13 different circulating lineages. The most common were B.1.1.70 and B.1.1.1, discovered in the majority of the samples (65.8%) (Figure 1). Following the GISAID/Nextrain nomenclature system, Serbian isolates were assigned into several clades. According to marker variants relative to the hCoV-19/Wuhan/WIV04/2019 (WIV04) reference, which are used by GISAID to classify isolates into different clades, sequences from this study were grouped into superclade G and clades GH, GR, and GV. The most dominant clade was GR (80.5%) (Figure 1). Clades S, L, and V were not detected. The third nomenclature system is Nextrain with its Nexclade tool (see text footnote 3) that uses clade-specific combinations of signature mutations. According to this system, the majority of sequences from this study were assigned to the 20D and 20B clades, which were dominant until September of 2020. The other clades that were detected in Serbian isolates were: 20A, 20A.EU2, and 20E (EU1) (Figure 1).

### Genetic Variability of Serbian SARS-CoV-2 Isolates

Compared to the reference Wuhan Hu-1 SARS-CoV-2 sequence, the Serbian SARS-CoV-2 whole genome sequences displayed 7–17 amino acid substitutions per isolate. There were 148 different genome variants (144 non-synonymous mutations, 2 nonsense, and 2 deletions) detected in the 41 isolates of the study. Variants were located within different regions of the viral genome. In Serbian SARS-CoV-2 isolates the most variable parts of the genome have been shown to be the following: nsp2, nsp3, nsp12, nsp13, nsp15, S protein, N protein, ORF3a, and ORF8 (Supplementary Table 1). It is known that the mutation rate is not similar for all SARS-CoV-2 proteins. WHO reported that the N protein had the highest percentage of variants considering the size of the protein (Koyama et al., 2020). In our study, N and S proteins were the most variable ones, with 21 and 23 different substitutions, respectively. Forty-five variants (45/148) were detected in two or more sequences. The analysis of identified variants in structural genes revealed that S and N genes were mutation hotspots. Almost one third (29.7%) of all variants were detected in these two genes. In the other group of non-structural genes, mutation hotspots were determined among genes encoding nsp3 and nsp12 (RNA-dependent RNA polymerase). Throughout the genome, two identical amino acid changes were conserved and found in all 41 isolates: Spike_D614G and nsp12_P323L. The next most commonly documented amino acid changes (in 32 isolates) were located in the N gene (N_R203K and N_G204R).

Ten of total 703 variants (1.41%) that passed the quality filters were indicative of heteroplasmy, but these were spread over 9 different individuals. Although the exact separation of true variants from sequencing errors in problematic regions needs further analysis, this distribution is indicative of a recent mutations and coexistence of both resulting strains rather than a co-infection with two separate strains.

According to comparison of the number of detected genome variants in Serbian isolates and global sequences from the GISAID database, variants were classified into four categories: unique (the first time detected in this study); extremely rare (detected in less than 0.01% of GISAID sequences published to date); rare (detected in less than 1% of GISAID published sequences); and common (often published in GISAID) (Supplementary Table 1). The unique mutation, detected for the first time in this study, was nsp12_V359L. The isolate with this unique mutation (C23860) was collected in October 2020. It belonged to clade 20E (EU1)/GV and lineage B.1.177. Among the Serbian isolates in the study period, there were four amino acid changes detected for the first time in Europe: nsp2_R246H, ORF3a_Y109H, ORF3a_D183N, and Spike_Y7070H (Supplementary Table 1).

Deletion of nine and six nucleotides was identified in two isolates, respectively. Isolate C17281 belonged to 20B/GR clade and the B.1.1.70 lineage contained a deletion in the nsp1 protein of three amino acids. In isolate L3072, a gap of six nucleotides (nt) led to deletions of ΔF263 and ΔE264 in nsp15. This six-nt gap has been reported in only seven sequences worldwide so far, according to the GISAID database as of February 2, 2021. Two stop codons were detected in ORF7a and ORF8 (Supplementary Table 1).

### Phylogenetic Position and Genetic Diversity of Serbian SARS-CoV-2 Isolates

**FIGURE 2:**
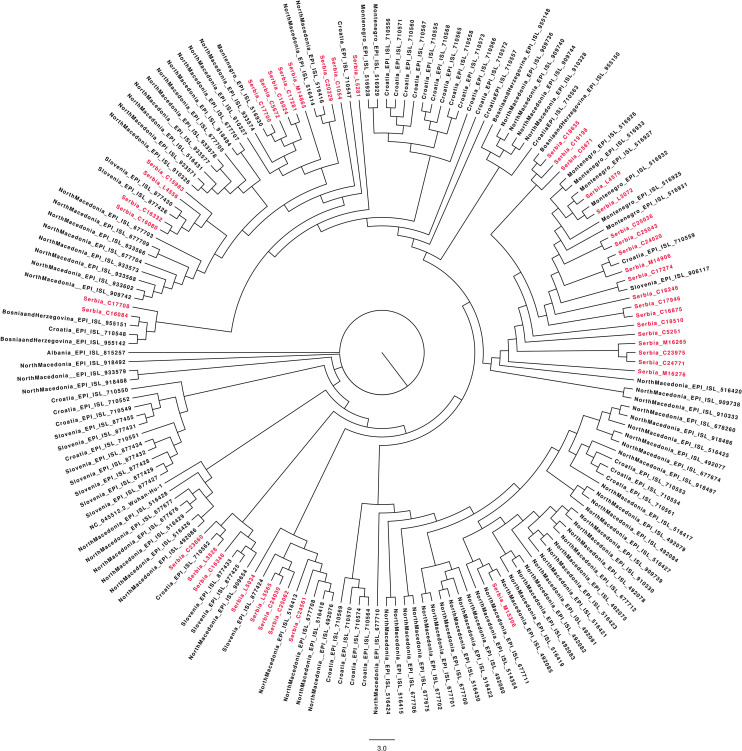
Maximum-likelihood (ML) phylogenetic tree generated by the W-IQ-TREE web server under the best-fit GTR^+^ G model of evolution, based on whole genome sequence of 41 Serbian SARS CoV2 isolates and all sequences from neighboring Balkan countries (Albania, Bosnia–Herzegovina, North Macedonia, Montenegro, Slovenia, and Croatia) from June–October 2020 were retrieved from GISAID database (*n* = 160). The reference sequence was Wuhan-Hu-1 NC 045512.2. The Serbian isolates are highlighted in the tree.

**FIGURE 3:**
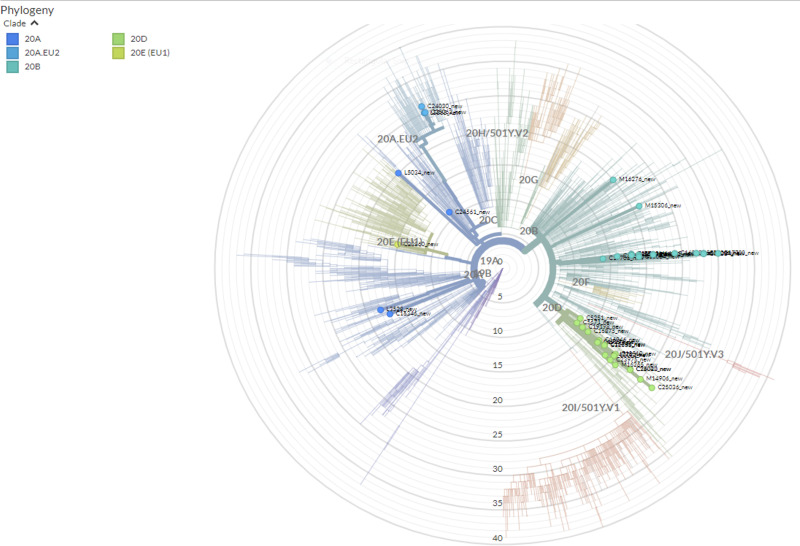
The phylogenetic tree of all SARS-CoV-2 isolates globally representing position and clade clustering (Nextrain nomenclature) of Serbian isolates of SARS CoV2 from the second wave of the pandemic. The tree was made online with Nextclade (https://clades.nextstrain.org/) without any restrictions on use.

A total of 41 full genome sequences were obtained from selected Serbian samples, which represent the second wave of SARS-CoV-2 pandemic in the country. Several clusters with Serbian isolates were observed using a maximum-likelihood phylogenetic tree (Figure 2). Some of those clusters contained only Serbian isolates and, in the others, Serbian isolates were grouped with isolates from Slovenia, North Macedonia, Croatia, Montenegro, and Bosnia–Herzegovina. The global comparison using the GISAID database and the Nexclade web-based application^7^ revealed that Serbian isolates from the second wave grouped into five clades (Figure 3).

## Discussion

In the last week of February 2020, the first nasopharyngeal and oropharyngeal swabs positive for SARS-CoV-2 started to be detected in Balkan countries. After the first registered case in Croatia, February 25, 2020, other countries in the region also began reporting their first cases of infection. Although it has only recently been established that the epidemic in Serbia existed even before the officially reported first case (Bogdanović et al., 2021), March 6, 2020 is considered as the official date of the beginning of the epidemic. In late spring and early summer 2020, Europe recorded a decline in the rate of infection and subsiding of the first wave. This trend lasted until the fall, when the second wave of the epidemic began to appear. Unlike the rest of Europe, Serbia was hit by the second wave of the epidemic a few months earlier. Already in June 2020, local confirmed cases had risen exponentially with up to nearly 900 newly registered cases daily which represents almost 0.5% of daily registered cases worldwide (Almubaid and Al-Mubaid, 2021). Summertime holidays, with the regional movement of tourists between Serbia and countries that had left borders open, had permitted expansion of the epicenter of the epidemic and made an impact on distribution dynamics of virus lineages and clades.

### Lineages and Clades

It has been demonstrated that whole genome sequencing of viruses during epidemics such as Ebola, Zika, and influenza represent a tremendous tool in tracing pathogen emergence and spread and also in monitoring transmission chains and viral evolution (du Plessis et al., 2021; Spanakis et al., 2021). The COVID-19 pandemic is the first pandemic in which there has been instant sharing of genomic information on isolates around the world, encouraging the reporting of the sequence variability dynamic from as many different local communities as possible (Hadfield et al., 2018). This strategy helps global molecular epidemiology networks to quickly, in real time, share up-to- date information about ongoing epidemics with the public, government and health organizations, and scientists. As a result, the rate of reported SARS-CoV-2 sequences has risen remarkably, with approximately 150,000 genomes shared online by the end of October 2020 on GISAID website.

The trademark variant of the G superclade is D614G in Spike protein. Globally, it was observed that between May and November 2020, the mutation D614G practically eliminated spread of other genome variations. This was also demonstrated in study sample of Serbian isolates during the summer wave. Other than that, the dominance of the GR clade (80.5%) in Serbian sequences was also in accordance with European clade distribution during the period June–September 2020. In agreement with Nextrain nomenclature the dominant clades until September 2020 were 20D and 20B. It should be noted that the most significant difference in the distribution of clades between Europe and Serbia involved 20D. This clade emerged over the summer of 2020, but lack of travel between European countries kept its frequency under 10% in Europe in the following months. On the other hand, the unique second wave in Serbia, in the same period, probably allowed the spread of 20D, so that this clade makes up approximately half of the clades in continuity. Summer holidays with regional travel possibly accelerated spreading of 20D. As a consequence, 20D was uniquely dominant in Balkan countries compared to the rest of Europe. However, during September and October, the local frequencies of clades changed, with the introduction of new clades [20A, 20A.EU2 and 20E (EU1)]. The influence of the momentum of the second European epidemic wave led to higher diversity. However, the 20D clade remained the most important characteristic of the local outbreak. Although in Europe at that time this clade was on the verge of elimination, the frequency of transmission in Serbia has been maintained.

Progressive introduction of new variants during the summer wave of the epidemic in Serbia, with increased diversity of variants in early autumn, was also demonstrated in the results of the lineage analysis. The only lineage that was circulating was lineage B, but 12 different sub-lineages were identified within it, most prominently sub-lineages B.1.1.1 and B.1.1.70. These two sub-lineages were introduced in Europe in early March 2020. In Serbia they were almost the only ones circulating until September 2020, when eight additional sub-lineages appeared just within a few weeks. Two isolates (C25036 and C25043) that were collected on October 13 belonged to C15 sub-lineage, also called Denmark lineage, which first appeared just a week before in Denmark (on October 5). In the phylogenetic tree they are grouped in the same clade.

### Variants Analysis

RNA viruses have a high mutation rate that often results in evolutionary advantage for better adaptation and survivability. Mutations lead to natural selection of those strains that show better viability, including drug resistance and immune evasion (Irwin et al., 2016). A complex of RNA- dependent RNA polymerase (RdRp) of SARS-CoV-2 and its cofactors nsp7 and nsp8 interacts with nsp14, making a unique proofreading mechanism during replication, which decreases the mutation rate of this coronavirus compared to that of other RNA viruses (Sevajol et al., 2014; Ferron et al., 2018). This leads to relatively slow mutation rate of 1–2 mutations per month along with the SARS-CoV-2 genome. Analysis of different local SARS-CoV-2 genomic surveillance revealed 3–22 mutations per genome in comparison to reference sequence (Mora et al., 2021) which is in line with our results (7–17 mutations per genome). Among 41 Serbian isolates 148 different genome variants were detected, of which the top four amino acid changes are also some of the most prominent amino acid changes worldwide (nsp12_P323L, Spike_D614G, N_R203K, and N_G204R). Furthermore, Serbian sequences revealed one unique amino acid substitution not previously registered.

We have found instances of heteroplasmy that corresponds to the published results (Hryhorowicz et al., 2020), but the somewhat smaller heteroplasmy rate is likely due to the more stringent quality filtration in this study.

#### Variants of ORF1ab

The main role of nsp2 protein is to interact with prohibitin (PHB) and prohibitin 2 (PHB2) in the host cell survival pathway (Rehman et al., 2020). Although this protein is not currently of particular importance to researchers, it is assumed that substitutions in the nsp2 domain could cause structural alterations, which might even influence the effect of antiviral therapeutics targeting the viral ORF1ab (Rehman et al., 2020). Among the different substitutions in nsp2 that were identified in Serbian isolates, one in particular attracted attention, namely, mutation R246H. This variant is extremely rare according to GISAID, as it has been found only in nine other sequences besides ours (in United States, Brazil, United Kingdom, Switzerland). It is also important that the Serbian isolate with this substitution was the first to be registered in Europe, 2 days before the sequence from the United Kingdom (August 4, 2020).

It is assumed that nsp3 works with nsp4 and nsp6 to induce activation of double-membrane vesicles (DMVs), which serve as an important part of the replication/transcription complex (Sakai et al., 2017). It is also well known that this gene has many variants. Accordingly, nsp3 has been shown to be highly variable, with 18 different substitutions in this research, too. The importance of evaluating all variants in nsp3 is that they affect the consequences of evolution of beta-coronaviruses by extensive selection pressure (Wu C. et al., 2020).

The protein nsp5, in all known coronaviruses, serves as the main protease for proteolytic processing of the polyprotein precursors (Roe et al., 2021). Along with nsp3 protease, which is known as an essential component for viral replication, it might represent a potential drug target. Two parts of the N terminal domain of nsp5 have been well characterized, but much less is known about the role of the alpha/helical domain of the protease. Analysis of isolates from our study showed diversity in four amino acid positions with the presence of the common substitution G15S found in almost half of our isolates. However, about a third of Serbian isolates also had the rare substitution G71S. Moreover, this mutation is a characteristic of the B.1.1.70 lineage and was never found together with the previously mentioned G15S. Finally, we identified an extremely rare P108T substitution which was reported only four times earlier in two European countries (United Kingdom and Slovenia), first in the United Kingdom in July and 3 months later in Serbia.

RNA-dependent RNA polymerase (RdRp) or nsp12 catalyzes RNA-template-dependent synthesis of phosphodiester bonds between ribonucleotides. As a key component of viral replication and transcription, it represents an important target for antiviral inhibitors such as favipiravir or remdesivir, which are some of the drugs used for the treatment of COVID-19 (Gordon et al., 2020). Because of that, substitutions in specific residues could lead in loss of drug efficacy. All Serbian isolates showed the P323L substitution (first reported in the United Kingdom in February 2020), which usually goes along with D614G substitution (Vilar and Isom, 2021). Although this substitution is not localized in the center of the catalytic site, there is a possibility that it could improve stability and intramolecular interactions in the protein and consequently affect the speed of viral replication (Chand et al., 2020; Khan et al., 2020). In the interface domain of the RdRp protein (A250-R365) where the P232L is located, in one Serbian isolate there was one more but unique substitution, V359L. As this variant was not reported earlier, it is important to register it, because all variants in this domain might have drastic impact on the function of RdRp as it assists in the coordination of N and C terminal domains of RdRp (Chand et al., 2020).

It was proposed that nsp15 is an endonuclease with protein activity responsible for the protein interference with the innate immune response and interferon-antagonizing properties (Deng et al., 2017; Yuen et al., 2020). In addition, even the nsp15 structure is very similar to the SARS-CoV and MERS-CoV homologs, and it shows some differences that may contribute to altered SARS-CoV-2 virulence (Kim Y. et al., 2020). Sequence analysis of Serbian isolates found a significant portion of T33I and V38F substitutions, but these two substitutions were never combined in the same isolates. Moreover, in one isolate that was sampled in October 2020, we identified an extremely rare deletion of six nucleotides (ΔF263 and ΔE264) that was the second registered European deletion, after Denmark, in March 2020. This deletion was also registered only seven additional times globally by the end of October 2020. The analysis of crystal structure of nsp15 of SARS-CoV-2 indicates that the metal binding site within main chain carbonyl oxygen atom of Pro263 could be required for maintaining conformation of the active site and substrate during catalysis (Kim Y. et al., 2020). Because of that, the consequence of the mentioned deletion should be further investigated.

#### Variants of the Spike Protein

The Spike protein of SARS-CoV-2 is a multifunctional molecule 1273 amino acids (aa) residue long, which binds to the ACE2 receptor during the viral entry into the host cell (Lan et al., 2020). It has differential forms of homotrimers projecting from the viral surface (Wrapp et al., 2020.) It contains two S1 and S2 subunits. Within S1, the receptor-binding domain (RBD) covers the segment from 319 to 541, with a loop region (424–494) that provides direct contact with the ACE2 receptor (Lan et al., 2020). In the S2 unit, heptad repeat domains (HR1 and HR2) interact to make closer contact between the virus and the entry point on the cell. An important component of the virus is also the furin cleavage site, located between the S1 and S2 subunits that facilitate the entry process. All the structures of the S protein form a six helix bundle (6HB) on the virus that facilitates the viral entry into the host cell.

One of the most dominant substitutions in SARS-CoV-2 is D614G in the Spike protein. It also represents the attributable substitution of the G superclade, and it has been linked to increased infectivity and transmissibility of SARS-CoV-2 (Brufsky, 2020; Korber et al., 2020). Taking this into account, in March 2020 it was noticed that the substitution showed a highly increased incidence in many regions of the globe, including the two Balkan countries that reported their sequences, Croatia and Bosnia–Herzegovina (Vilar and Isom, 2021). In line with the global gradual loss of variants without D614G substitution, all Serbian sequences from the second wave of the epidemic had this substitution.

Mutations of the RBD domain residues have attracted differential approaches for analysis of possible consequences of those mutations. At the same time, investigations include sequence diversity, not only because of receptor binding activity, but also because of the current spread of new variants or discovery of feasible drugs and vaccines that could inhibit the virus host entry. There is a vast amount of genome sequencing data that conclude that the single amino acid substitution is directly proportional to the functional contacts of surface protein and receptor (Schreiner et al., 2011). For example, substitution N501Y, which increases binding capacity to ACE2 receptor and many more substitutions (K417N, K417T, E484K etc.) that characterize three major variants of concern (VOC) (B.1.1.7, P.1, and B.1.351.), was not found in Serbian isolates up to October 2020. However, three isolates from this study showed the rare substitution L452R, which turned out to be a characteristic of the newly identified variants B.1.427 and B.1.429. More importantly, those two variants were recently (in March 2021) classified in the group of variants of concern because they affect receptor binding affinity, with transmission increased by 20% and reduction in neutralization using convalescent and post-vaccination sera (Deng et al., 2021). This finding implies a relatively long and multi-month circulation of a combination of mutations that later become the main feature of VOC.

Finally, the extremely rare substitution Y707H was the second found worldwide (after United States), but the first found in European sequences. The location of this mutation is within the S2 fusion subunit, with a yet unknown consequence for the function of this region.

#### Variants of the N Protein

The N protein is a structural protein that self-oligomerizes to encapsulate RNA and binds to multiple sites within SARS-CoV-2 genome (Chang et al., 2014). N protein is 419 amino acids (aa) long and is divided into three domains: N terminal domain (NTD 46–176 aa), C terminal domain (CTD 247–364 aa) and long intrinsically disordered region (IDR 187–247 aa) (Kang et al., 2020; Liang et al., 2020; Zeng et al., 2020). Part of the IDR, which is called serine/arginine-rich segment (SR domain 183–206 aa), is crucial for oligomerization (He et al., 2004) and is capable of modulating viral infectivity via nsp3-N protein interaction (Hurst et al., 2013). The nucleocapsid interacts with the replication and transcription complex and in that way influences the replication performance of SARS-CoV-2 (Taylor et al., 2009; Verheije et al., 2010).

The N protein is highly immunogenic; high levels of IgG antibodies against N have been detected in sera from SARS patients and are plentiful during infection (Leung et al., 2004; Cong et al., 2020). The analysis of Serbian isolates from this study revealed that the N protein was harboring the most variants after S protein. In our study of the SR-rich domain, some of the detected substitutions (S194L, S202C, and T205I) may lead to loss of phosphorylation sites (serines and threonines), can induce additional flexibility of the IDR, expose some new sites for hydrophobic interaction, and possibly increase susceptibility for proteolysis (Garvin et al., 2020). The most frequent amino acid substitutions, detected in 78% of Serbian SARS-CoV-2 isolates, were R203K and G204R within the SR-rich domain of the N protein. These double substitutions appear at high frequencies (more than 150,000 sequences of the N protein published so far) and it is a very common substitution in this SR-rich domain of N protein globally. Amino acids R and K are both polar and relatively large, so replacement at position 203 is conservative. On the other hand, G204R results in an additional polar positively charged site, and it may induce increased local rigidity (Garvin et al., 2020). The possible explanation for positive selection of this double mutation may be because these changes do not occlude phosphorylation sites, impact oligomerization, or expose the protein backbone to proteolysis (Garvin et al., 2020). In the N and C terminal domain of N protein, 2 and 11 different variants were detected in this study, respectively. The amino acids at positions 101–120, 156–170, 266–280, and 361–370 in the N protein of SARS-CoV-2 are T-cell epitopes and recognized by CD4 T cells (Le Bert et al., 2020; Nelde et al., 2021.) Changes at positions D103, P168, P279, and P368 detected in this study have been reported to be related to antigenic drift (Le Bert et al., 2020; Nelde et al., 2021). Three isolates from this study had the same pattern of substitutions in C terminal domain of N protein (M234I, A376T, and R385K), but these amino acid replacements were conservative and should not impact any viral feature, but additional functional characterization is needed to understand role of these amino acid changes detected in different part of the N protein.

#### Variants of the Open Reading Frame 3a (ORF3a)

ORF3a is a 275-amino acid protein that consists of the N terminal, transmembrane, and C terminal domains (Bianchi et al., 2021). ORF3a protein contains domains that have different functions: an ion channel promoting viral release, caveolin-binding domain that regulates the viral cycle, and TRAF3-binding motif (TRAF–TNF receptor-associated factors) that induces apoptosis and inflammatory response in the infected cells (Lu et al., 2006; Padhan et al., 2007; Scott and Griffin, 2015; Siu et al., 2019; Issa et al., 2020). The most frequent amino acid change was Q57H, identified in 12.2% of Serbian SARS-CoV-2 isolates. This is in accordance with the literature in which Q57H is continuously the most dominant ORF3a substitution (Liu et al., 2020). Although this site is located within the transmembrane part of the ORF3a, this amino acid substitution did not impose any functional changes (Nelson et al., 2020; Bianchi et al., 2021). Two extremely rare substitutions (Y109H and D183N) detected in this study in ORF3a are the first such amino acid changes registered among European isolates. Residue C133 is a conserved site of ORF3a among different species and has a role in homodimerization (Bianchi et al., 2021). One sample in our study harbored two extremely rare substitutions C133S and Y109H where radical replacement of polarity and volume of amino acid took place. Further functional characteristics are needed in order to understand any possible impact of these new and rare substitutions.

#### Variants of the E and M Gene and Open Reading Frames (ORF6, ORF7, and ORF8)

The E and M ORF6 and ORF7 are less susceptible to variants than other parts of the SARS-CoV-2 genome. The envelope protein, smallest among structural proteins, consists of three domains: N terminal, transmembrane, and C terminal domains (Hassan et al., 2020). The DLLV motif within the C-terminal domain binds to tight junction-associated protein (PALS1) to ease the way for infection (Teoh et al., 2010). In our study we found only one amino acid substitution in the E protein in one SARS-CoV-2 isolate. This substitution, L73F, is s rare and changes the DLLV motif to DFLV that may influence infectivity and/or replication of the virus (Hassan et al., 2020). M glycoprotein spans the membrane bilayer, can bind to all other structural proteins, and is important for budding process (Mousavizadeh and Ghasemi, 2021). M protein is conserved between coronaviruses and only two different variants (A38S and V60L) were identified in this study, in three isolates. Replacement of A residue by S and V by L is conservative; it did not affect charge, polarity, and volume of amino acids involved, so this change probably will not affect function of the M protein in any way.

ORF6, ORF7, and ORF8 are accessory proteins that are key players in blocking the innate immune response *in vivo*, suppressing IFN production and signaling pathways (Frieman et al., 2007). In Serbian SARS-CoV-2 isolates several amino acid changes were detected within these proteins with low frequency. The only substitution with higher frequency (10%) was V62L in ORF8.

The limitations of this study include a small sample size and incomplete epidemiological and clinical data. In order to derive final conclusions about COVID-19 epidemic in Serbia and reconstruct the possible routes for the spread of particular clades, further sequencing is needed. Nevertheless, as our findings are the first from the region that is undersampled, they provide a significant contribution to national and global understanding of SARS-CoV-2 genetic diversity.

## Conclusion

We provide a comprehensive analysis of SARS-CoV-2 sequences in the unique early second wave of the European epidemic. This analysis of Serbian isolates represents the first publication from Balkan countries, which demonstrates the importance of specificities of local transmission especially when preventive measures differ among countries. One unique and seven extremely rare variants were identified, with locally specific continuous dominance of the 20D clade. At the same time, some of the trademark changes of newly identified variants of concern were found in our isolates from October 2020. Future research should be focused on functional characterization of novel variants in order to understand the exact role of these variations.

## Data Availability Statement

All the sequences obtained in this study are deposed in GISAID database (https://www.gisaid.org) under following accession numbers: EPI_ISL_891266 - EPI_ISL_891272, EPI_ISL_909737, EPI_ISL_918411, EPI_ISL_1055778, EPI_ISL_1055779, EPI_ISL_1055782, EPI_ISL_1055783, EPI_ISL_1055786, EPI_ISL_1055787, EPI_ISL_1055790, EPI_ISL_1055791, EPI_ISL_1055793, EPI_ISL_1055794, EPI_ISL_1057035, EPI_ISL_1057036, EPI_ISL_1057038, EPI_ISL_1057040, EPI_ISL_1057041, EPI_ISL_1057097, EPI_ISL_1057098, EPI_ISL_1056970, EPI_ISL_1056972, EPI_ISL_1056974, EPI_ISL_1056956, EPI_ISL_1056957, EPI_ISL_1056959, EPI_ISL_1056968, EPI_ISL_1057102, EPI_ISL_1057103, EPI_ISL_1057104, EPI_ISL_1057105, EPI_ISL_1061425, EPI_ISL_1061427, EPI_ISL_1061429, EPI_ISL_1061430, and EPI_ISL_1061431.

## Ethics Statement

The studies involving human participants were reviewed and approved by the Ethical Board of the Institute for Biocides and Medical Ecology, Belgrade, Serbia. The patients/participants provided their written informed consent to participate in this study.

## Author Contributions

AL and DD coordinated collection and processing the clinical samples. AL performed RNA isolation and RT-PCR. DA coordinated NGS. OM performed bioinformatics analysis of NGS isolates and participated in manuscript writing. DM and AB analyzed all the data, performed phylogenetic analysis, and wrote the manuscript. AB conceived and coordinated the study. All authors contributed to the manuscript editing and approved the final version.

## Conflict of Interest

The authors declare that the research was conducted in the absence of any commercial or financial relationships that could be construed as a potential conflict of interest.

## Footnotes

1 https://www.gisaid.org/

2 https://pangolin.cog-uk.io/

3 https://clades.nextstrain.org/

4 https://www.gisaid.org/epiflu-applications/covsurver-mutations-app/

5 http://iqtree.cibiv.univie.ac.at/

6 http://tree.bio.ed.ac.uk/software/figtree/

7 https://clades.nextstrain.org/tree
