# Supplementary material for: The First Molecular Characterization of Serbian SARS-CoV-2 Isolates From a Unique Early Second Wave in Europe
Source: Front Microbiol. 2021 Jun 18;12:691154. doi: 10.3389/fmicb.2021.691154 (PMC8250835; doi:10.3389/fmicb.2021.691154)
Supplement: Supplementary file 1 [file Table_1.DOC]

Supplementary Table1

**Table 1.** Variant analyses of SARS-CoV-2 genome among 41 Serbian Covid-19 cases

| **Location in SARS-CoV-2 protein** | **Variant** | **Type** | **Number (%) of samples in Serbian study** | **Variant’s frequency categories***** (compared to the sequences from GISAID)** |
| --- | --- | --- | --- | --- |
| **nsp1** | V86F | Non-synonymous | 1 (2.43) | Rare |
|  | E93K | Non-synonymous | 2 (4.87) | Rare |
|  | K141del | Deletion | 1 (2.43) | Rare |
|  | S142del | Deletion | 1 (2.43) | Rare |
|  | F143del | Deletion | 1 (2.43) | Rare |
| **nsp2** | T44I | Non-synonymous | 1 (2.43) | Rare |
|  | M141V | Non-synonymous | 1 (2.43) | Rare |
|  | R246H | Non-synonymous | 1 (2.43) | Extremely rare  Note: The registered mutation is the first among European isolates.  Reported nine times globally. |
|  | A249V | Non-synonymous | 1 (2.43) | Rare |
|  | A306V | Non-synonymous | 1 (2.43) | Rare |
|  | M551I | Non-synonymous | 1 (2.43) | Rare |
|  | P624L | Non-synonymous | 1 (2.43) | Rare |
| **nsp3** | A85V | Non-synonymous | 1 (2.43) | Rare |
|  | S126P | Non-synonymous | 1 (2.43) | Rare |
|  | E195K | Non-synonymous | 3 (7.32) | Rare |
|  | P340S | Non-synonymous | 1 (2.43) | Rare |
|  | E374A | Non-synonymous | 1 (2.43) | Rare |
|  | E391D | Non-synonymous | 1 (2.43) | Rare |
|  | E374A | Non-synonymous | 1 (2.43) | Rare |
|  | S403P | Non-synonymous | 1 (2.43) | Rare |
|  | T428I | Non-synonymous | 19 (46.34) | Rare |
|  | M494I | Non-synonymous | 1 (2.43) | Rare |
|  | T749I | Non-synonymous | 1 (2.43) | Rare |
|  | T819I | Non-synonymous | 1 (2.43) | Rare |
|  | M829I | Non-synonymous | 1 (2.43) | Rare |
|  | K1077N | Non-synonymous | 14 (34.17) | Rare |
|  | A1183V | Non-synonymous | 4 (9.76) | Rare |
|  | M1278I | Non-synonymous | 1 (2.43) | Rare |
|  | D1283Y | Non-synonymous | 1 (2.43) | Rare |
|  | S1717L | Non-synonymous | 1 (2.43) | Rare |
| **nsp4** | M324I | Non-synonymous | 3 (7.32) | Rare |
|  | A380V | Non-synonymous | 3 (7.32) | Rare |
|  | A446V | Non-synonymous | 2 (4.87) | Rare |
| **nsp5** | G15S | Non-synonymous | 18 (43.90) | Common |
|  | G71S | Non-synonymous | 13 (31.70) | Rare |
|  | P108T | Non-synonymous | 1 (2.43) | Extremely rare  Note: Reported four times in two European countries. |
|  | A260V | Non-synonymous | 1 (2.43) | Rare |
| **nsp6** | H11Y | Non-synonymous | 2 (4.87) | Rare |
| **nsp7** | K70N | Non-synonymous | 4 (9.76) | Rare |
| **nsp8** | A27V | Non-synonymous | 1 (2.43) | Rare |
|  | T145I | Non-synonymous | 1 (2.43) | Rare |
| **nsp9** | T35I | Non-synonymous | 3 (7.32) | Rare |
|  | Q49H | Non-synonymous | 1 (2.43) | Rare |
| **nsp10** | R134H | Non-synonymous | 1 (2.43) | Rare |
| **nsp12** | K91R | Non-synonymous | 1 (2.43) | Rare |
|  | A185S | Non-synonymous | 3 (7.32) | Common |
|  | P227S | Non-synonymous | 1 (2.43) | Rare |
|  | R249S | Non-synonymous | 2 (4.87) | Rare |
|  | P323L | Non-synonymous | 41 (100) | Common |
|  | V359L | Non-synonymous | 1 (2.43) | Unique |
|  | D454E | Non-synonymous | 1 (2.43) | Rare |
|  | V776L | Non-synonymous | 3 (7.32) | Common |
|  | S913L | Non-synonymous | 4 (9.76) | Rare |
| **nsp13** | K218R | Non-synonymous | 3 (7.32) | Common |
|  | E261D | Non-synonymous | 3 (7.32) | Common |
|  | V356F | Non-synonymous | 1 (2.43) | Rare |
|  | A368S | Non-synonymous | 1 (2.43) | Rare |
|  | D466Y | Non-synonymous | 1 (2.43) | Rare |
|  | T481M | Non-synonymous | 1 (2.43) | Rare |
|  | P504S | Non-synonymous | 1 (2.43) | Rare |
| **nsp14** | Y235F | Non-synonymous | 1 (2.43) | Rare |
|  | E365K | Non-synonymous | 1 (2.43) | Rare |
|  | L366F | Non-synonymous | 1 (2.43) | Rare |
|  | L493F | Non-synonymous | 1 (2.43) | Rare |
|  | V510F | Non-synonymous | 1 (2.43) | Rare |
| **nsp15** | V10L | Non-synonymous | 4 (9.76) | Rare |
|  | T33I | Non-synonymous | 13 (31.70) | Rare |
|  | V38F | Non-synonymous | 15 (36.58) | Rare |
|  | T48I | Non-synonymous | 1 (2.43) | Rare |
|  | T112I | Non-synonymous | 1 (2.43) | Rare |
|  | F263del  E264del | Deletion | 1 (2.43) | Extremely rare  Note: The registered 6-nt deletion is the second among European isolates. It has been reported  seven times globally. |
|  | P270S | Non-synonymous | 1 (2.43) | Rare |
| **nsp16** | T140I | Non-synonymous | 2 (4.87) | Rare |
|  | A188S | Non-synonymous | 1 (2.43) | Rare |
| **Spike** | A67V | Non-synonymous | 1 (2.43) | Rare |
|  | H69Y | Non-synonymous | 1 (2.43) | Rare |
|  | R78G | Non-synonymous | 1 (2.43) | Rare |
|  | S98F | Non-synonymous | 1 (2.43) | Common |
|  | R214H | Non-synonymous | 1 (2.43) | Rare |
|  | D215Y | Non-synonymous | 1 (2.43) | Rare |
|  | A222V | Non-synonymous | 1 (2.43) | Common |
|  | A262S | Non-synonymous | 2 (4.87) | Common |
|  | T299I | Non-synonymous | 1 (2.43) | Rare |
|  | E309Q | Non-synonymous | 2 (4.87) | Rare |
|  | Q314K | Non-synonymous | 1 (2.43) | Rare |
|  | D427G | Non-synonymous | 1 (2.43) | Rare |
|  | L452R | Non-synonymous | 3 (7.32) | Rare |
|  | S477N | Non-synonymous | 3 (7.32) | Common |
|  | A520S | Non-synonymous | 1 (2.43) | Rare |
|  | D614G | Non-synonymous | 41 (100) | Common |
|  | A684V | Non-synonymous | 1 (2.43) | Rare |
|  | V687I | Non-synonymous | 1 (2.43) | Rare |
|  | Y707H | Non-synonymous | 1 (2.43) | Extremely rare  Note: The registered mutation is the only one among European isolates, and the second reported globally. |
|  | G769V | Non-synonymous | 1 (2.43) | Rare |
|  | A1078S | Non-synonymous | 1 (2.43) | Rare |
|  | P1162R | Non-synonymous | 1 (2.43) | Rare |
|  | V1264L | Non-synonymous | 2 (4.87) | Rare |
| **ORF3a** | G11R | Non-synonymous | 1 (2.43) | Rare |
|  | T34A | Non-synonymous | 4 (9.76) | Rare |
|  | Q38R | Non-synonymous | 1 (2.43) | Common |
|  | Q57H | Non-synonymous | 5 (12.19) | Common |
|  | L106F | Non-synonymous | 2 (4.87) | Rare |
|  | Y109H | Non-synonymous | 1 (2.43) | Extremely rare  Note: The registered mutation is the only among European isolates. It has been reported 19 times globally. |
|  | C133S | Non-synonymous | 1 (2.43) | Extremely rare  Note: Reported globally nine times. |
|  | R134H | Non-synonymous | 1 (2.43) | Rare |
|  | T151I | Non-synonymous | 1 (2.43) | Rare |
|  | S171L | Non-synonymous | 1 (2.43) | Rare |
|  | G172R | Non-synonymous | 1 (2.43) | Rare |
|  | D183N | Non-synonymous | 1 (2.43) | Extremely rare  Note: The registered mutation is the only one among European isolates. |
|  | K192E | Non-synonymous | 1 (2.43) | Rare |
|  | V202L | Non-synonymous | 1 (2.43) | Common |
| **E protein** | L73F | Non-synonymous | 1 (2.43) | Rare |
| **M protein** | A38S | Non-synonymous | 1 (2.43) | Rare |
|  | V60L | Non-synonymous | 2 (4.87) | Rare |
| **ORF6** | H3Y | Non-synonymous | 1 (2.43) | Rare |
|  | I26V | Non-synonymous | 1 (2.43) | Rare |
| **ORF7a** | T28I | Non-synonymous | 1 (2.43) | Rare |
|  | F87L | Non-synonymous | 1 (2.43) | Rare |
| **ORF7b** | E3stop | Nonsense | 1 (2.43) | Rare |
| **ORF8** | G8R | Non-synonymous | 1 (2.43) | Rare |
|  | V62L | Non-synonymous | 4 (9.76) | Rare |
|  | D63G | Non-synonymous | 1 (2.43) | Rare |
|  | E106stop | Nonsense | 1 (2.43) | Rare |
|  | L118V | Non-synonymous | 1 (2.43) | Rare |
|  | F120L | Non-synonymous | 2 (4.87) | Rare |
|  | I121L | Non-synonymous | 2 (4.87) | Rare |
| **N protein** | D103Y | Non-synonymous | 1 (2.43) | Rare |
|  | P168S | Non-synonymous | 1 (2.43) | Rare |
|  | S194L | Non-synonymous | 1 (2.43) | Common |
|  | P199Q | Non-synonymous | 1 (2.43) | Rare |
|  | P199L | Non-synonymous | 1 (2.43) | Common |
|  | G200S | Non-synonymous | 1 (2.43) | Rare |
|  | S202C | Non-synonymous | 1 (2.43) | Rare |
|  | R203K | Non-synonymous | 32 (78.05) | Common |
|  | G204R | Non-synonymous | 32 (78.05) | Common |
|  | T205I | Non-synonymous | 1 (2.43) | Common |
|  | G215V | Non-synonymous | 1 (2.43) | Rare |
|  | A220V | Non-synonymous | 1 (2.43) | Common |
|  | M234I | Non-synonymous | 3 (7.32) | Common |
|  | G238C | Non-synonymous | 3 (7.32) | Rare |
|  | P279S | Non-synonymous | 1 (2.43) | Rare |
|  | P368L | Non-synonymous | 2 (4.87) | Rare |
|  | D371N | Non-synonymous | 1 (2.43) | Rare |
|  | A376T | Non-synonymous | 3 (7.32) | Common |
|  | D377Y | Non-synonymous | 1 (2.43) | Common |
|  | R385K | Non-synonymous | 3 (7.32) | Rare |
|  | A414S | Non-synonymous | 2 (4.87) | Rare |

***unique** - the first time detected in this study

**extremely rare** - detected in less than 0.01% of GISAID sequences published to date

**rare** - detected in less than 1% of GISAID published sequences

**common** - often published in GISAID
